# Association between increased anterior cingulate glutamate and psychotic-like experiences, but not autistic traits in healthy volunteers

**DOI:** 10.1038/s41598-023-39881-1

**Published:** 2023-08-07

**Authors:** Verena F. Demler, Elisabeth F. Sterner, Martin Wilson, Claus Zimmer, Franziska Knolle

**Affiliations:** 1 Department of Diagnostic and Interventional Neuroradiology, Klinikum rechts der Isar, Technical University of Munich, Ismaninger Straße 22, 81675 Munich, Germany; 2 Centre for Human Brain Health and School of Psychology, University of Birmingham, Birmingham, UK; 3 Department of Psychiatry, University of Cambridge, Cambridge, UK

**Keywords:** Translational research, Neuroscience

## Abstract

Despite many differences, autism spectrum disorder and schizophrenia spectrum disorder share environmental risk factors, genetic predispositions as well as neuronal abnormalities, and show similar cognitive deficits in working memory, perspective taking, or response inhibition. These shared abnormalities are already present in subclinical traits of these disorders. The literature proposes that changes in the inhibitory GABAergic and the excitatory glutamatergic system could explain underlying neuronal commonalities and differences. Using magnetic resonance spectroscopy (^1^H-MRS), we investigated the associations between glutamate concentrations in the anterior cingulate cortex (ACC), the left/right putamen, and left/right dorsolateral prefrontal cortex and psychotic-like experiences (Schizotypal Personality Questionnaire) and autistic traits (Autism Spectrum Quotient) in 53 healthy individuals (26 women). To investigate the contributions of glutamate concentrations in different cortical regions to symptom expression and their interactions, we used linear regression analyses. We found that only glutamate concentration in the ACC predicted psychotic-like experiences, but not autistic traits. Supporting this finding, a binomial logistic regression predicting median-split high and low risk groups for psychotic-like experiences revealed ACC glutamate levels as a significant predictor for group membership. Taken together, this study provides evidence that glutamate levels in the ACC are specifically linked to the expression of psychotic-like experiences, and may be a potential candidate in identifying early risk individuals prone to developing psychotic-like experiences.

## Introduction

Already in the earliest clinical reports of schizophrenia and autism spectrum disorder (ASD), their commonalities and associations have been discussed^1–3^. On a phenomenological level, schizophrenia and ASD overlap regarding negative symptoms, social cognition, and perceptual alterations^4^. Additionally, both disorders display similar cognitive deficits in terms of working memory function, perspective taking, or response inhibition^5^. Nevertheless, ASD and schizophrenia show distinct clinical profiles and clinical progressions^6,7^. While ASD is a neurodevelopmental disorder with an early onset, usually diagnosed in childhood, and is characterized by a stable or improving long-term prognosis^8^, schizophrenia typically develops later in adolescence or early adulthood and is associated with persistent long-term impairment^9^. The greatest difference between the disorders, however, lies within the type of positive symptoms: while individuals with ASD may show repetitive or restricted behaviors, individuals with schizophrenia may suffer from hallucinations or delusions^10^.

Despite those differences, both disorders share common environmental risk factors, genetic predispositions as well as neuronal abnormalities^4,7^. Patients with ASD and schizophrenia show, for example, abnormal development in the anterior cingulate cortex (ACC), striatum and frontal lobe^11–13^. Additionally, shared atypical connections were identified in the default mode network and the salience network^14^. Both disorders show similar alterations in reward processing^15,16^ and prediction error learning^17–20^, with individuals with ASD showing greater impairment in social reward and prediction error learning. Importantly, both disorders show substantial overlap already at the subclinical and trait level^21–23^.

Potential candidates providing an explanation for the underlying neuronal commonalities and differences are different neurotransmitter systems such as the glutamatergic system. The glutamate hypothesis of schizophrenia is well described^24,25^, building on the discovery that antagonists of a glutamate receptor (N-methyl-d-aspartate (NMDA) receptor) induced psychotic symptoms. Alterations in neurotransmitter systems may be assessed non-invasively with magnetic resonance spectroscopy (MRS). A recent review^24^ summarizes that glutamate and Glx (glutamate + glutamine) concentrations are increased in the basal ganglia, thalamus and medial temporal lobe in schizophrenia patients. However, other studies reveal more inconsistent findings, showing increased prefrontal glutamate in anti-psychotic naïve patients^26^, while another study^27^ reports glutamate reductions in the ACC in psychosis, but could not detect alterations in the putamen or DLPFC. Reductions in glutamate concentrations have been found more consistently in the ACC in antipsychotic-naïve early psychosis patients^28^, medicated early psychosis patients^29^ and a mix of early psychosis and chronic schizophrenia patients^30^. This supports the theory that increased glutamate is a sign of an acute psychotic or prodromal phase which builds up to a first episode and is linked to inflammatory processes^31,32^. Interestingly, there are strong links between altered levels of glutamate and neurofunction, which are task-dependent^33^. In a recent systematic review, Zahid and colleagues^34^ reported reduced positive associations between ACC glutamate levels and brain activity during resting state conditions, but increased positive associations between ACC glutamate levels and brain activity during cognitive control tasks in early psychosis patients. Wenneberg and colleagues^35^ and Bojesen and colleagues^36^ found an association between glutamate and GABA levels and clinical symptoms and cognition in ultra-high-risk individuals in cortical and subcortical regions, supporting the association between ACC glutamate and cognition in the pathophysiology of early psychosis. Together these studies seem to suggest that there are complex interactions, dependent on the brain state, between cortical and subcortical glutamate that may be altered in schizophrenia.

Also, in ASD research there is emerging evidence that an imbalance between different neurotransmitter systems, mainly the excitatory glutamatergic and inhibitory GABAergic system, contributes to the development of the disorder. Alterations with respect to glutamate have been reported in many cortical and subcortical regions. Horder and colleagues^37^ for example found reduced levels of glutamate in the striatum to be associated with the severity of social symptoms in ASD. Interestingly, Page and colleagues^38^ found increased concentrations of glutamate/glutamine in the amygdala/hippocampus, but not in parietal regions, while others found increased glutamate in the inferior frontal gyrus^39^, the sensory motor cortex (children^40^) or the putamen^41^. Another study^42^ found reduced glutamate concentrations in the auditory cortex and increased concentrations in the ACC. Alterations in these regions have also been reported by others^43,44^. However, also in ASD findings are inconsistent^45–47^. Both disorders are associated with changes in cortico-striatal-thalamic circuits^48–50^ and abnormal excitatory glutamate neurotransmitter concentrations in overlapping cortical and subcortical areas^27,37,51–55^ including the cingulate cortex, the prefrontal cortex and striatum. This suggests that a complex interaction of altered levels of glutamate in different cortical and subcortical regions may contribute to the development of symptoms within those two disorders.

Consistent with the continuum model of psychotic disorders, such as schizophrenia, and ASD, symptoms of schizophrenia and autism are reflected on a spectrum ranging from subclinical variants of psychotic-like experiences and autistic traits to clinical states of schizophrenia and ASD respectively^56^ where schizophrenia and autism are seen as extremes of a continuum diverging in opposite directions from normality^6,57^. Frameworks of subclinical traits allow the investigation of neurobiological mechanisms underlying symptomatology without the influence of potential confounders such as medication, illness duration or age onset. Traits of these disorders may be assessed with the Schizotypal Personality Questionnaire (SPQ) and the Autism Spectrum Quotient (AQ) for psychotic-like experiences and autistic traits, respectively. As for the clinical stages of the disorders, strong associations between both subclinical spectra are reported in the literature. A recent meta-analysis^22^ revealed a high correlation between autistic and global psychotic-like experiences of 0.48.

Only few studies explored changes of neurotransmitter concentrations associated to psychotic-like experiences and autistic traits. Ford and colleagues^58^ investigated whether abnormalities in the glutamate/GABA ratio were linked to symptoms shared between the two traits and symptom severity using MRS. They found a positive correlation between glutamate/GABA ratio in the right superior temporal gyrus and the total scores of AQ, SPQ and AQ + SPQ. Additionally, positive correlations with subscales related to social skills and communication^58^, indicating that alterations in glutamatergic and GABAergic neurotransmitter systems are associated with shared symptoms. Given that the literature reports alterations of neurotransmitter concentrations, mainly glutamate, across a number of different brain regions, it is likely that those regions contribute in an interactive way to the development of clinical symptoms.

The aim of the present study was, therefore, to use MRS to explore if and how glutamate concentrations in five cortical and subcortical areas interact and are associated with autistic traits and psychotic-like experiences in a sample of healthy individuals. The regions included the ACC, the left and right dorsolateral prefrontal cortex (DLPFC), and the left and right putamen. We hypothesized that increased levels of glutamate in the ACC and reduced levels of glutamate in the DLPFC and putamen would be associated with psychotic-like experiences. On the other hand, we hypothesized that reduced levels of ACC glutamate and increased levels of glutamate in the putamen would be associated with autistic traits.

## Methods

### Participants and subclinical questionnaires

53 healthy subjects (26 women) aged 18–35 years participated in this study. See supplementary materials for recruitment and inclusion criteria. The study was approved by the medical research ethics committee of the Technical University of Munich. All subjects gave written informed consent in accordance with the Declaration of Helsinki.

All participants completed a German version^59^ of the Schizotypal Personality Questionnaire (SPQ)^60^ capturing psychotic-like experiences; as well as the Autism Spectrum Quotient (AQ)^61^ assessing autistic traits (Table 1, Fig. S1; see supplementary material for details on correlations between AQ and SPQ).

**Table 1:** Demographic data and clinical scores.

	Female (n = 26)	Male (n = 27)	P-value^1^	W
Age	23.31 (3.54)	23.93 (4.21)	0.7266	331
SPQ: total score (/296)	97.00 (45.54)	84.26 (52.36)	0.2547	415
AQ: total score (/50)	21.65 (6.99)	20.96 (7.65)	0.7017	373

Values are mean (SD).

SPQ, Schizotypal Personality Questionnaire; AQ, Autism Spectrum Quotient.

^1^Wilcoxon rank sum test.

### MR Image acquisition

**Figure 1:**
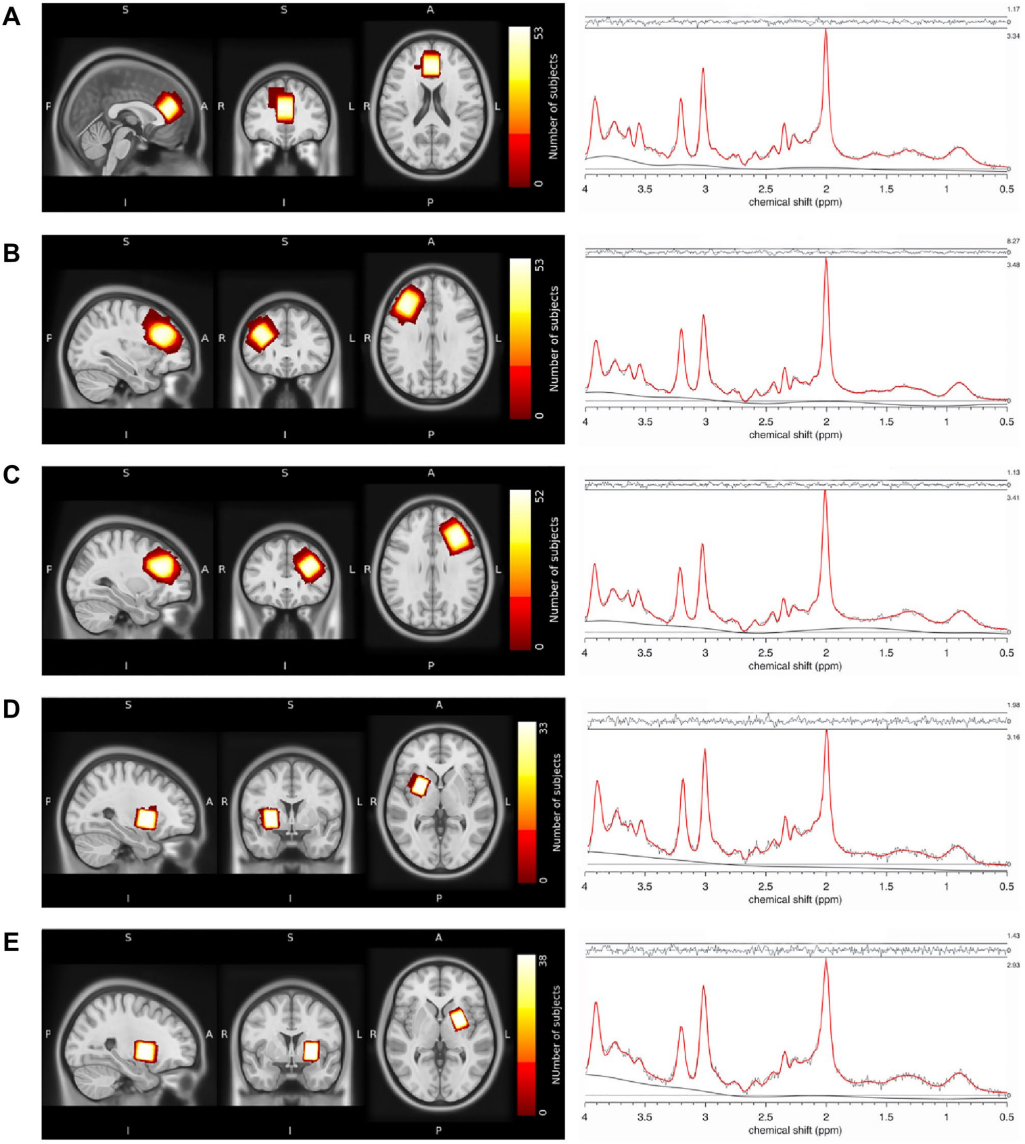
Voxel placement and representative fitted MRS spectra. Placement of MRS voxels and ^1^H-MRS spectrum fitted by Osprey of the (**A**) ACC, (**B**) DLPFC right, (**C**) DLPFC left, (**D**) putamen right, and (**E**) putamen left. The colors indicate the areas covered by the subjects’ individually placed MRS voxels. The individual voxels were standardized with SPM12, overlapped in MRIcroGL and visualized in FSLeyes. ACC, anterior cingulate cortex; DLPFC, dorsolateral prefrontal cortex.

Structural MRI and ^1^H-MRS data were collected on a 3 T Philips Ingenia Elition X MR-Scanner (Philips Healthcare, Best, The Netherlands) using a 32-channel head coil. For anatomical reference and spectroscopic voxel placement, we acquired a T1-weighted magnetization prepared rapid gradient echo (MPRAGE) sequence: echo time (TE) = 4 ms, repetition time (TR) = 9 ms, Flip angle (α) = 8°, shot interval = 3000 ms, slice number = 170, matrix size = 240 × 252 and voxel size = 1 × 1 × 1 mm^3^. MRS data were collected using a ^1^H-MRS single voxel ECHO volume Point Resolved Spectroscopy Sequence (PRESS) sequence: TR = 2000 ms, TE set to shortest (35.6–41.2 ms), samples = 1024, bandwidth = 2000 Hz, phase cycle steps = 16, and flip angle = 90°. We used the conventional Philips water suppression technique (excitation) that performs Automatic Water Suppression Optimization (AWSO) pre-scans to minimize the residual water (window = 140 Hz, second pulse angle = 300). See supplementary materials for further descriptions. We placed one voxel in the ACC (20 × 20 × 20 mm) and one voxel per hemisphere in the Putamen (20 × 15 × 20 mm) as well as in the DLPFC (30 × 20 × 20 mm); see Fig. 1A–E for voxel placement overlap for each of the five regions.

### ^1^H-MRS analysis

The MRS data were analyzed using Osprey version 2.4.0, an all-in-one software for state-of-the-art processing and quantitative analysis of in-vivo MRS data^62^. As eddy-current correction had already been performed during the automatic pre-processing of the scanner, this was excluded from the automatic preprocessing in Osprey. Subsequently, Osprey executed the remaining processing steps appropriate to the provided data, including frequency-and-phase alignment, water removal, frequency referencing, and initial phasing.

As the TE of our MRS data varied between and within the individual voxels to ensure the shortest acquisition time, we simulated six different PRESS basis sets for each TE (36, 37, 38, 39, 40, 41) with a bandwidth of 2000 Hz and 1024 pts using MARSS in INSPECTOR version 11-2021^63^. Based on recommendations in the LCModel manual^64^, we selected the following metabolites: alanine, aspartate, creatine (Cr), GABA, glucose, glutamate (Glu), glutamine, glutathione, glycerophosphocholine, lactate, myoinositol, NAA, N-acetyl-aspartylglutamate (NAAG), phosphocholine, phosphocreatine (PCr), scyllo-inositol and taurine. To this basis set we added the default macromolecular and lipid components provided by Osprey.

To analyze our data in Osprey, we utilized the LCModel^65^ implementation for fitting and quantification. The T1-weighted images were segmented in grey matter (GM), white matter (WM), and cerebrospinal fluid (CSF) using SPM12 within Osprey. The processing followed standard parameters, with a metabolite fit range of 0.5 to 4.0 ppm and a water fit range of 2.0 to 7.4 ppm. The knot spacing used was 0.4 ppm. Finally, Osprey estimates the tissue and relaxation corrected molal concentration according to the Gasparovic method^66^. See supplementary materials for a detailed workflow diagram (Supplementary Materials Fig. S2A).

Osprey provides data quality outcomes, including signal-to-noise ratio (Cr SNR; ratio between amplitude of Cr peak and standard deviation of detrended noise in the range of − 2 to 0 ppm^62^), linewidth for water [full-width half-maximum (FWHM) of single-Lorentzian fit to H2O reference peak^62^] and Cramer-Rao lower bounds (CRLB) for each estimated metabolite. All spectra were visually inspected for artifacts. An exemplary fit of the spectra can be seen in Fig. 1A–E. Spectral exclusion criteria were therefore either visual failure of the fitting algorithm, a resultant FWHM > 13 Hz in the ACC and DLPFC or FWHM > 10 Hz in the putamen, with thresholds chosen according to recommendations for B0-shimming provided by Juchem et al.^67^, or a CRLB > 20% of glutamate concentration.

### ^1^H-MRS glutamate levels and spectral quality

We measured the tissue and relaxation corrected molal concentration of glutamate (mol/kg) in five voxels of interest, the ACC, the left putamen, right putamen, left DLPFC and right DLPFC. For the ACC and the right DLPFC, the fit of the glutamate spectra were appropriate for all participants. For the left DLPFC, one subject had to be excluded due to visual failure of the fit. Although the visual inspection was appropriate, but generally showing more variation in the residuals, for the left and right putamen, 15 subjects from the left putamen and 20 from the right putamen were excluded due to FWHM > 10 Hz. Results are presented in Table 2. Due to the substantial loss of data in the putamen, we did not include glutamate levels derived from the putamen in any of the following analysis.

**Table 2:** ^1^H-MRS quality parameters and glutamate levels by region.

Region	ACC	PUT R	PUT L	DLPFC R	DLPFC L
n	53	33	38	53	52
Glu (SD), mol/kg	28.11 (1.91)	25.69 (1.90)	25.56 (1.96)	20.83 (1.86)	21.28 (1.99)
CRLB (SD), %	5.25 (0.52)	5.85 (0.76)	6.08 (0.78)	5.72 (0.57)	5.73 (0.60)
SNR (SD)	101.06 (13.88)	48.65 (5.21)	47.23 (6.74)	126.77 (19.74)	124.14 (15.75)
FWHM (SD), Hz	6.18 (0.44)	9.18 (0.48)	9.05 (0.53)	7.55 (0.68)	7.71 (0.74)
GM vol (SD), %	69.21 (4.07)	70.26 (5.18)	68.57 (4.65)	43.03 (5.55)	41.21 (5.97)
WM vol (SD), %	11.54 (2.74)	29.53 (5.12)	31.20 (4.66)	50.99 (8.23)	53.13 (7.12)
CSF vol (SD), %	19.25 (3.21)	00.21 (0.60)	00.23 (0.44)	5.98 (3.82)	5.66 (1.91)

Values are mean (SD).

ACC, anterior cingulate cortex; PUT R, right putamen; PUT L, left putamen; DLPFC R, right dorsolateral prefrontal cortex; DLPFC L, left dorsolateral prefrontal cortex; Glu, glutamate; CRLB, Cramer-Rao Lower Bound; SNR, signal-to-noise ratio; FWHM, full width at half maximum, measurement for the water linewidth; GM, grey matter; WM, white matter; CSF, cerebrospinal fluid; vol, volume; SD, standard deviation.

### Association between glutamate and clinical scores

To understand the contributions of glutamate concentrations in different cortical regions to symptom expression, we first conducted two linear regression models with psychotic-like experiences and autistic traits as outcome variables and levels of glutamate derived from the ACC, left DLPFC and right DLPFC as predictor variables. Furthermore, we corrected for age and sex.

**Figure 2:**
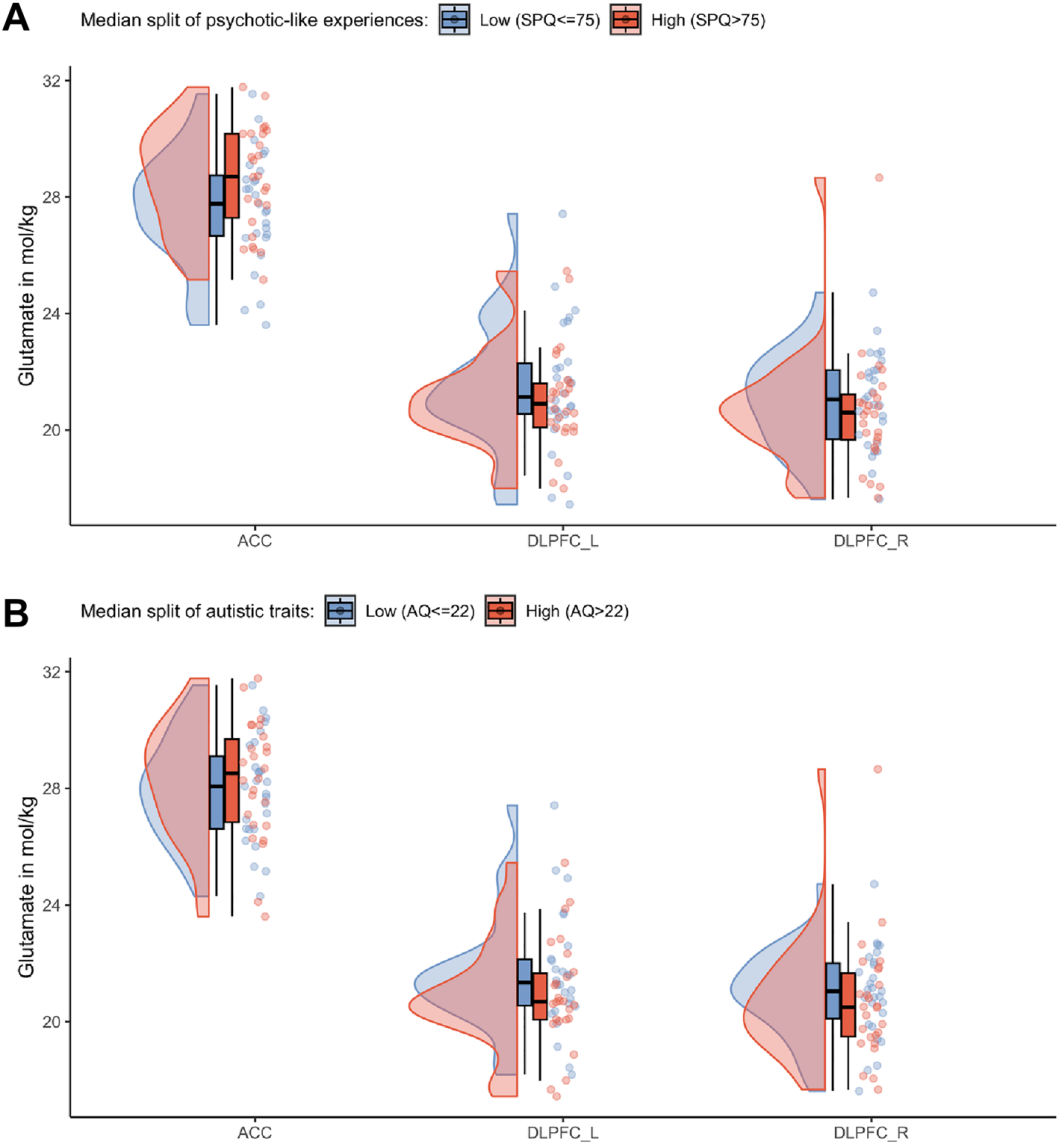
Glutamate distribution after a median split of SPQ and AQ. Differences of the glutamate levels in the ACC, left and right DLPFC after a (**A**) Median split of psychotic-like experiences with a median of SPQ = 75. (**B**) Median split of autistic traits with a median of AQ = 22.

To investigate non-linear relationships between clinical traits and glutamate concentrations, we dichotomized the clinical scores and performed logistic regression analyses. This is important as there has been criticism of the hypothesis of a continuous development of neurobiological changes in psychosis from sub-threshold symptoms to severe psychotic symptoms^20,68,69^. To dichotomize the clinical scores, we performed a median split, defining a high and low risk group separately for psychotic-like experiences (median = 75) and autistic traits (median = 22). See Fig. 2 for distributions of glutamate by group, and Supplementary Table S3 for demographic and clinical differences. Using a logistic regression, we investigated whether any predictor variables (i.e., levels of glutamate derived from the ACC, left DLPFC and right DLPFC) significantly contributed to a change in the response variable (i.e., low vs high psychotic-like experiences) from 0 to 1 (i.e., from low to high psychotic-like experiences). Also, the logistic regressions were corrected for age and sex. The significance level was set to p < 0.05. Bonferroni multiple comparison corrections were applied, corrected significance levels are reported together with the results. Analyses were performed in R using the stats package version 4.0^70^.

## Results

### Association between psychotic-like experiences and glutamate concentration in the anterior cingulate cortex

**Figure 3:**
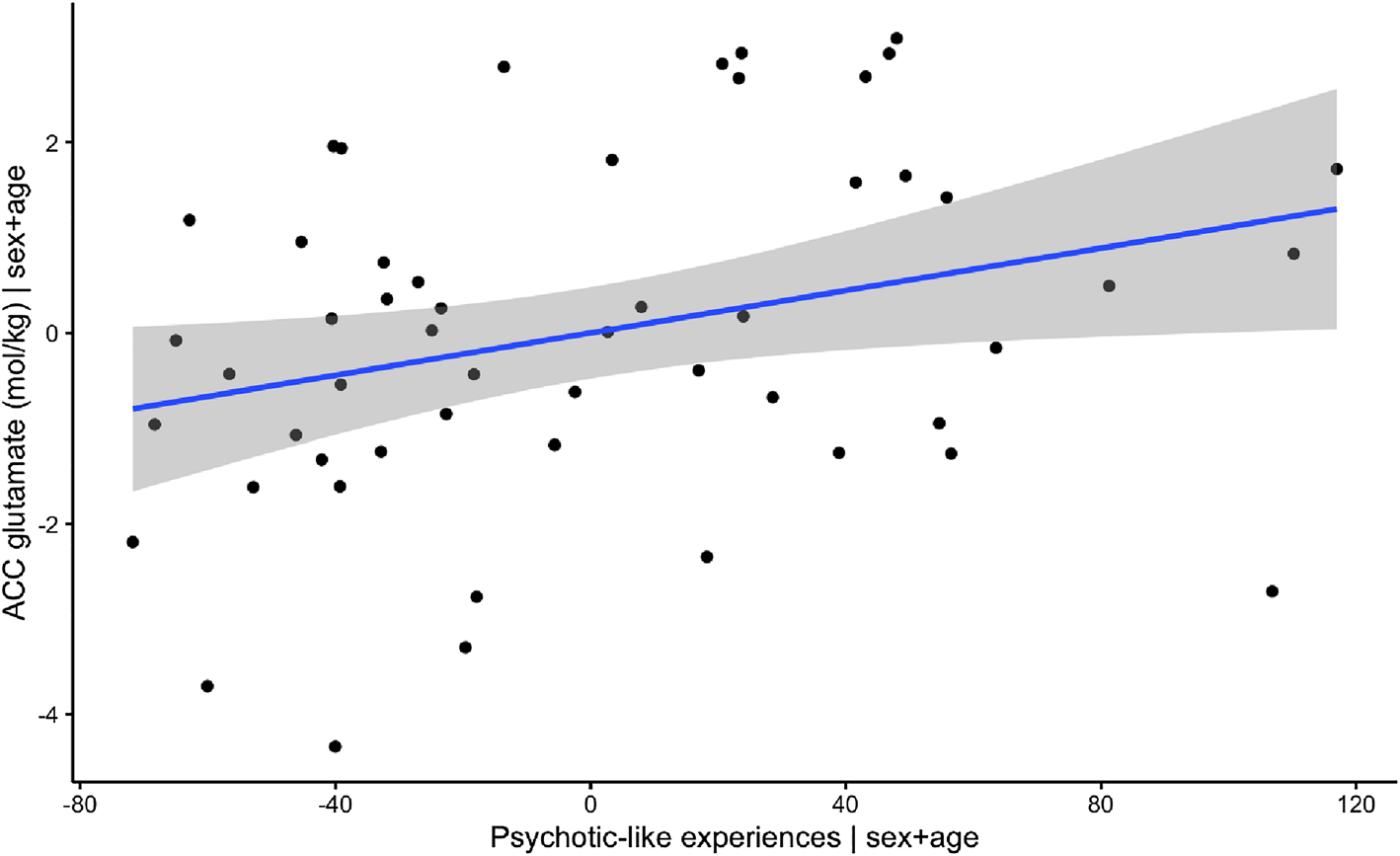
Visualization of association between levels of glutamate in ACC and psychotic-like experiences, when correcting for age and sex.

We first fitted two multiple linear regression models to test if absolute glutamate concentrations in the ACC, left DLPFC and right DLPFC predicted symptom scores. The first fitted regression model was: psychotic-like experiences ~ Glu DLPFC_R + Glu DLPFC L + Glu ACC + age + sex. The overall regression was not significant (R2 = 0.15, F(5,46) = 1.63, p = 0.17). Importantly, however we found that levels of glutamate in the ACC significantly predicted psychotic-like experiences (β = 9.72, 95% CI [2.17, 17.27] p = 0.013) (Fig. 3). Autistic traits however were not predicted by levels of glutamate.

Supporting this finding, the binomial logistic regression (PLE-group ~ Glu DLPFC_R + Glu DLPFC L + Glu ACC + age + sex) revealed that while holding all other predictor variables constant, the odds of an individual belonging to the high psychotic-like experiences group increased by 33.1% (β = 0.49, 95% CI [0.01, 2.70], p = 0.014) for a one-unit increase in ACC glutamate. We did not see this effect of glutamate on autistic traits. The effects of the linear and logistic regressions both survived Bonferroni multiple comparison correction (level of significance, p < 0.025, correcting for two tests each). As many studies use Glx (glutamate + glutamine) to measure glutamate, we computed the same analysis using Glx, and replicated our results, which are presented in the Supplementary Materials. Also see Supplementary Table S1 for an overview on levels of Glx. Furthermore, we also computed the same analysis using a novel RStudio based MRS analysis tool—spant^71^. Again, the results are similar, although only marginally significant, and are presented in the Supplementary Materials, together with a detailed description of the method and analysis (Supplementary Table S2, Supplementary Figs. S2B, S3).

## Discussion

In this study, we explored if and how glutamate concentrations in the ACC and left and right DLPFC and putamen are associated with autistic traits and psychotic-like experiences in a sample of healthy individuals. We found that glutamate concentrations in the ACC predicted psychotic-like experiences in healthy individuals, but they did not predict autistic traits. Supporting this finding, we furthermore found that glutamate concentrations in the ACC contributed significantly to the determination of group status (i.e., low psychotic-like experiences vs high psychotic-like experiences) when applying a median split to the clinical data. This was not the case for autistic traits. Regarding the glutamate concentrations in the DLPFC and the putamen, however, we found that whereas the DLPFC did not yield any significant results, levels of glutamate could not be reliably estimated in the putamen.

### Association between ACC glutamate and psychotic-like experiences

ACC glutamate levels have been associated with psychotic-like experiences in healthy individuals^72^, symptoms and structural changes in high-risk individuals^73^, symptoms in first episode psychosis^73–75^ and chronic psychosis^76^. Importantly, our results provide support for both a dimensional and a non-linear relationship, showing a positive linear relationship between the severity of psychotic-like experiences and ACC glutamate across the complete sample; importantly however, ACC glutamate is also predictive of whether or not an individual classifies as a high-risk versus a low-risk individual, indicating qualitative differences in the neuropathology of the different disease stages^69^. This however needs to be explored in future research.

In general, evidence on altered levels of ACC glutamate in the schizophrenia spectrum is inconsistent with some studies reporting higher levels^31,74^, while others find reductions^29,30^ or no differences^77,78^. Although Modinos and colleagues^78^ did not find different levels of glutamate in the ACC between individuals with high schizotypy and low schizotypy (schizotypy and psychotic-like experiences have overlapping meanings, and are two concepts to describe the liability for psychosis^79,80^), they found that increased grey matter volume in ACC was negatively related to ACC glutamate. This supports the view that alterations in glutamatergic levels may explain structural changes which are associated with the development of psychotic-like experiences. The association in this, but also in other studies^72^, however, suggests that decreasing levels of glutamate in the ACC are associated with specific pathologies in psychosis, whereas our study indicates that higher levels of ACC glutamate are associated with the development of psychotic-like experiences. Our results correspond to findings from Demro and colleagues^81^ who reported that increased subclinical symptoms of grandiosity were linked to increased levels of glutamate in the ACC. Egerton and colleagues^82^ found higher levels of glutamate/creatine-ratio in the ACC in symptomatic first episode psychosis patients compared to those in remission. In a different study, Egerton and colleagues^83^ found that higher levels of glutamate metabolites in the ACC predicted treatment response, indicating that higher baseline levels are associated with poorer treatment response, while lower levels of ACC glutamate are predictive of improvements across positive and negative symptoms as well as general functioning in first episode psychosis patients. Relatedly, Godlewska and colleagues^27^ reported lower levels of glutamate in the ACC in early psychosis patients compared to healthy controls. Importantly, however, the majority of patients included in this study were medicated, which confirms results from a recent meta-analysis^84^ on the impact of anti-psychotic treatment on frontal glutamate levels. The authors found that antipsychotics were linked to a significant decrease in frontal Glx levels in both first episode and chronic schizophrenia patients, with the effect being stronger in first episode psychosis patients. As our results indicate that ACC glutamate levels are predictive of individuals belonging to the high psychotic-like experience group, the results are in line with the theory that increased glutamate is a sign of an acute psychotic or prodromal phase which builds up to a first episode of psychosis and is linked to inflammatory processes^31,32^. The theory further suggests that a hypofunction of NMDA receptors reduces the activity of inhibitory GABAergic interneurons, on which they are located. This activity reduction increases glutamatergic neurotransmission of pyramidal cells, and may contribute to the development of, especially, positive symptoms during an acute episode of psychosis^32,85^.

### Lack of effect for other regions and autistic traits

Surprisingly, our data did not reveal any associations between the DLPFC and psychotic-like experiences. Generally, the literatures present an inconsistent picture regarding glutamate concentration in different regions. For example, although Godlewska and colleagues^27^ reported alterations in glutamate in the ACC in early psychosis patients compared to healthy controls, they did not find differences in the putamen and DLPFC. However, especially the literature with regard to subclinical schizotypal traits is sparse, and mainly concentrates on ACC and hippocampal glutamate^72,78^. To our knowledge, only one other study investigated prefrontal glutamate in individuals with high schizotypy compared to low schizotypy^86^, in which the authors reported a reduction of glutamate in high schizotypy.

Furthermore, our data did not reveal any associations between glutamate concentrations and autistic traits despite several studies showing an interaction, which is at odds with results reported in patients suffering from ASD (e.g.,^37,38,40,42–45^). Interestingly, studies exploring psychotic-like experiences and autistic traits together found similar patterns between both subclinical scores. For example, results by Ford and colleagues^87^ suggest that social disorganization is associated with increased glutamate/GABA + ratio in the right superior temporal region across both the autistic and schizotypal spectrum. Whereas, Kondo and colleagues^88^ report that autistic and schizotypal traits were associated with the Glutamine + Glutamate/GABA ratio in the auditory cortex but not in the frontal areas, including ACC, DLPFC and inferior frontal cortex.

Based on these studies and the fact that psychotic-like experiences and autistic traits are highly correlated, this might not be surprising at all. Our results however clearly indicate no such relationship between glutamate and autistic traits. We hypothesize that the relationship between changes in glutamate and autistic traits is mediated by psychotic-like experiences, which is rooted in their high correlation. To provide some initial evidence we computed an exploratory non-parametric causal mediation model, which is described in the supplementary materials. Indeed, our results revealed that the association between ACC glutamate and autistic traits is fully mediated by psychotic-like experiences. This may suggest that the high correlation between the two subclinical concepts results has a confounding on the relationship, and that underlying and often unassessed psychotic-like experiences may be the driving factor in the relationship between glutamatergic changes and autistic traits. However, more research is needed to explore this hypothesis.

### Consistency of results using different measures of glutamate and a novel toolbox

In addition to our analysis using the LCModel implementation in Osprey and extracting absolute water and tissue corrected levels of glutamate (mol/kg), we also replicated our results using Glx as well as a novel r-implemented toolbox (spant^71^). The replication of our results using different types of concentrations and methods further increases confidence in our results. Nevertheless, studies should focus on consistent ways of measuring metabolite differences, as it is not only the choice of location for voxel placement that differs grossly among studies but also the choice of metabolite representation (e.g., glutamate/GABA ratio, absolute glutamate, glutamate/creatine ratio, etc.) and selection of basis sets.

## Limitations

This study has several limitations. First, we did not analyze levels of GABA in the voxels of interest, as no spectral editing was applied to the MRS sequence. Future studies should try to measure both metabolites reliably in order to understand interactions between neurotransmitters and regions. Second, due to high values of FWHM, glutamate concentrations estimated in the left and right putamen had to be excluded from the analysis. One possible explanation for this is that subcortical voxels are usually noisier. Higher resolution (e.g., 7 T) or spectral editing of the sequence should be applied to improve data quality. Third, we were unable to recruit participants with highly increased scores along both spectra. One possible explanation is that many individuals with highly increased scores would already have a clinical psychiatric diagnosis, which was an exclusion criterion in our study. Nevertheless, follow-up studies should recruit larger samples allowing for a larger spectrum. Fourth, our sample does not include many individuals with low SPQ and high AQ scores, and vice versa, which would be necessary in order to clearly differentiate between clinical traits. Yet, our sample represents the distribution of subclinical symptoms within the general population as confirmed by similar correlations of the two scores compared to previous studies^22,89^. Bigger samples would be favorable to also investigate the clinical subscores (e.g., positive-like experiences, negative-like experiences).

## Conclusion

Taken together, this study shows that changes in glutamate in the ACC are associated with the manifestation of psychotic-like experiences but not autistic traits, which indicates that an imbalance in the glutamatergic neurotransmitter system involving the ACC may contribute to the development of psychotic-like experiences specifically. These results suggest that glutamate levels could potentially serve as a promising indicator for identifying individuals at an early stage who are susceptible to experiencing psychotic-like episodes.

### Supplementary Information

Supplementary Information.

## Data Availability

Data is available upon reasonable request to the corresponding author (franziskaknolle@gmail.com/franziska.knolle@tum.de).
