# Supplementary material for: Association between increased anterior cingulate glutamate and psychotic-like experiences, but not autistic traits in healthy volunteers
Source: Sci Rep. 2023 Aug 7;13:12792. doi: 10.1038/s41598-023-39881-1 (PMC10406950; doi:10.1038/s41598-023-39881-1)
Supplement: Supplementary file 1 — Supplementary Information. [file 41598_2023_39881_MOESM1_ESM.docx]

**Running title:** Glutamate, schizotypal and autistic traits

**Association between increased anterior cingulate glutamate and psychotic-like experiences, but not autistic traits in healthy volunteers**

Demler et al.

**Methods**

**Participants**

53 healthy participants (26 women, 27 men) aged between 18 to 35 years were recruited through local advertisements and social media. To evaluate eligibility for participating in our study at the Technical University of Munich, all subjects completed a clinical online questionnaire to assess their psychotic-like experiences and autistic traits. Demographic data and medical history were collected during a brief telephone screening to ensure the fulfillment of our inclusion criteria. All participants were German native speakers; right-handed; had no diagnosis of schizophrenia, psychosis or autism, or any neurological disease or injury and were currently not taking any psychoactive medication or had a change in their medication within at least the past six weeks. All participants had no contraindications for MRI scanning. Three subjects had a previous diagnosis of depression, one of them with an additional diagnosis of attention deficit disorder, two had previous eating disorders, one was diagnosed as schizoid with adjustment disorder and another subject reported a post-traumatic stress disorder and a narcissistic personality disorder. However, only one of these participants was taking a stable dose of medication for attention deficit disorder and depression (fluoxetine, atomoxetine). Details of demographic data and symptom scores are shown in Table 1 and Figure S1. The study was approved by the medical research ethics committee of the Technical University of Munich. All subjects gave written informed consent in accordance with the Declaration of Helsinki.

### **Schizotypal Personality Questionnaire**

The Schizotypal Personality Questionnaire (SPQ) is a widely used 74-item self-report questionnaire to capture psychotic-like experiences according to the DSM-III-R based constructs of schizotypal personality disorder symptoms ^1^. It measures nine dimensions of psychotic-like experiences which can be assigned to a three-factor structure consisting of positive-like symptoms (i.e., ideas of reference, magical thinking, unusual perceptual experiences, paranoia), negative-like symptoms (i.e., no closer friends, constricted affect, social anxiety), and disorganized traits (i.e., odd speech, odd behavior; ^2^). In its original version, the SPQ is administered in a forced-choice yes/no. Here we applied a German translation ^3^ of the modified version of the SPQ using a 5-point Likert scale version (strongly disagree=0, disagree=1, neutral=2, agree=3, strongly agree=4) with a maximum of 296 points, which increases the sensitivity of detecting schizotypy traits ^4^.

### **Autism Spectrum Quotient**

The Autism Spectrum Quotient (AQ) is an established 50-item self-report questionnaire which is designed to assess five different facets of autistic spectrum traits (social skill, attention switching, attention to detail, communication, imagination) ^5^. The AQ is administered in a 4-point Likert scale format (definitely agree, slightly agree, slightly disagree, definitely disagree). For the evaluation, a binary scoring method is used with the presence of autistic traits, either mildly or strongly, generating one point while the opposite is scored zero, leading to a maximum score of 50. The items are counterbalanced so that half of the items are worded to produce a disagree response and the other half to produce an agree response in a high scoring individual. In the present study, we applied a German translation of the AQ scale.

**The association between autistic traits and schizotypal traits**

To investigate the relationship between the SPQ subscales and autistic traits, we calculated Spearman's rank correlation coefficients as the assumptions of normality, linearity and homoscedasticity were not met. The correlation analyses were performed in R using the Hmisc package version 4.7.1 (https://hbiostat.org/R/Hmisc/).

**^1^H-MRS Analysis with spant**

The MRS data were also analyzed using Spectroscopy Analysis Tools (spant) version 2.6.9 ^6^ (<https://martin3141.github.io/spant/index.html>), an open-source R toolbox. The fitting in spant uses an adaptive baseline fitting algorithm (ABfit) ^7^, which accurately estimates the optimal baseline as it is known that the level of baseline smoothness plays a crucial role as an analysis parameter in metabolite estimation. Further spant integrates the strengths of R with a combination of traditional and modern MRS data processing techniques, making it well-suited for fully automated routine MRS analysis ^6^.

As our data was automatically pre-processed by the scanner using eddy-current correction, we only applied two more steps on our data: the first one was water removal, which eliminated the residual water signal with a Hankel singular value decomposition (HSVD) filter ^8^; and the second was phase-frequency alignment, which aligned the spectrum to the total N-acetylaspartate (NAA) resonance at 2.01 ppm.

We used SPM12 ^9^ to segment the structural T1 image into the tissue fractions - grey matter (GM), white matter (WM), and cerebrospinal fluid (CSF). We then ran the ABfit ^7^ fitting method of spant with the provided tissue fraction parameters to quantify glutamate. We used the tissue and relaxation corrected molal glutamate concentration based on Gasparovic and colleagues ^10^ for our further analyses. See supplementary materials for a detailed workflow diagram (Figure S2B).

Spant provides data quality outcomes, including signal-to-noise ratio (SNR; defined as the maximum signal value divided by the standard deviation of the noise ^6^), linewidth for tNAA and Cramer-Rao lower bounds (CRLB) for each estimated metabolite. All spectra were visually inspected for artefacts. An exemplary fit of the spectra can be seen in Figure S3. Spectral exclusion criteria were therefore either visual failure of the fitting algorithm, a resultant tNAA linewidth > 0.1 ppm ^11^ or a CRLB > 20% of glutamate concentration.

**1H-MRS glutamate levels and spectral quality for spant+ABfit**

We measured the tissue and relaxation corrected molal concentration of glutamate in five voxels of interest, the ACC, the left putamen, right putamen, left DLPFC and right DLPFC. For the ACC and the right DLPFC the fit of the glutamate spectra were appropriate for all participants. For the left DLPFC, two subjects had to be excluded due to visual failure of the fit. Although the visual inspection was appropriate, but generally showing more variation in the residuals, for the left and right putamen, one subject from the left putamen and three from the right putamen were excluded due to tNAA linewidth > 0.1ppm. Results are presented in Table S2. Due to better comparability to the results in Osprey+LCM, we did not include glutamate levels derived from the putamen in any of the following analysis.

**Results**

**Correlation between the SPQ and AQ**

Using the Spearman's rank correlation coefficient, we found a moderate, significant correlation between psychotic-like experiences and autistic traits (r=0.51, p<0.001). Correlation strength was classified according to Akoglu ^12^. The correlations between the subscores are shown in Figure S4. The chord diagram was created in R using the circlize-package Version 0.4.15 ^13^.

**^1^H-MRS Glx levels and spectral quality**

At 3 T, it is difficult to distinguish between Glu and Gln, so they are usually measured together as Glx (the signal of both glutamate and glutamine combined) ^14^. Some studies nevertheless prefer glutamate as the output value. So, we measured both concentrations in our five voxels of interest, the ACC, the left/right putamen, and the left/right DLPFC. Results are presented in Table S1.

**Association between psychotic-like experiences and Glx concentration in the anterior cingulate cortex**

We first fitted two multiple linear regression models to test if absolute glutamate concentrations in the ACC, left DLPFC and right DLPFC predicted symptom scores. The first fitted regression model was: psychotic-like experiences ~ Glx DLPFC_R + Glx DLPFC L + Glx ACC + age + sex. The overall regression was not significant (R2 = 0.11, F(5,46) = 1.114, p = 0.37). Importantly, however we found that levels of glutamate in the ACC significantly predicted psychotic-like experiences (β = 7.65, p = 0.042). Autistic traits however were not predicted by levels of glutamate.

Supporting this finding, the binomial logistic regression (PLE-group ~ Glx DLPFC_R + Glx DLPFC L + Glx ACC + age + sex) revealed that while holding all other predictor variables constant, the odds of high psychotic-like experiences occurring increased by 16.9% (coeff. est.=0.43, 95% CI [0.08, 2.33], p=0.024) for a one-unit increase in ACC glutamate. We did not see this effect of glutamate on autistic traits**.**

**Association between psychotic-like experiences and Glu concentration in the anterior cingulate cortex measured with spant+ABfit**

We first fitted two multiple linear regression models to test if absolute glutamate concentrations in the ACC, left DLPFC and right DLPFC predicted symptom scores. The first fitted regression model was: psychotic-like experiences ~ Glx DLPFC_R + Glx DLPFC L + Glx ACC + age + sex. The overall regression was not significant (R2 = 0.09, F(4,46) = 1.124, p = 0.35). Similarly as for the results derived by Osprey, we found that levels of glutamate in the ACC predicted psychotic-like experiences with a marginal significance (β = 4.61, p = 0.053). Autistic traits however were not predicted by levels of glutamate.

Also, the binomial logistic regression (PLE-group ~ Glx DLPFC_R + Glx DLPFC L + Glx ACC + age + sex) revealed similar results namely that while holding all other predictor variables constant, the odds of high psychotic-like experiences occurring increased, with marginal significance, by 48.9% (coeff. est.=0.18, 95% CI [0.01, 1.49], p=0.079) for a one-unit increase in ACC glutamate. We did not see this effect of glutamate on autistic traits**.**

**Explorative mediation analysis of psychotic-like experiences mediating the association between ACC glutamate and autistic traits**

We set up an explorative causal mediation model investigating the mediating effect of psychotic-like experiences on the association of ACC glutamate on autistic traits, using the r-package “mediation” ^15^ and applying non-parametric bootstrapping, to provide evidence for why other studies find associations between levels of glutamate and autistic traits, while we lack this finding. Unstandardized indirect effects were computed for each of 5000 bootstrapped samples, and the 95% confidence interval was computed by determining the indirect effects at the 2.5th and 97.5th percentiles. The fitting assumptions (global stat, skewness, kurtosis, link function, heteroscedasticity) were all acceptable.

The results of this mediation analysis revealed that the effect of ACC glutamate on autistic traits was fully mediated via psychotic-like experiences. The bootstrapped unstandardized indirect effect was 0.59, and the 95% confidence interval ranged from 0.02 to 1.23. Thus, the indirect effect was statistically significant (p=0.04).

**References**

1 Raine A. The SPQ: A Scale for the Assessment of Schizotypal Personality Based on DSM-III-R Criteria. *Schizophr Bull* 1991; **17**: 555–564.

2 Wuthrich V, Bates TC. Confirmatory Factor Analysis of the Three-Factor Structure of the Schizotypal Personality Questionnaire and Chapman Schizotypy Scales. *J Pers Assess* 2006; **87**: 292–304.

3 Klein C, Andresen B, Jahn T. Erfassung der schizotypen Persönlichkeit nach DSM-III-R: Psychometrische Eigenschaften einer autorisierten deutschsprachigen Übersetzung des ‘Schizotypal Personality Questionnaire’ (SPQ) von Raine. [Psychometric assessment of the schizotypal personality according to DSM-III-R criteria: Psychometric properties of an authorized German translation of Raine’s ‘Schizotypal Personality Questionnaire’ (SPQ).]. *Diagnostica* 1997; **43**: 347–369.

4 Wuthrich V, Bates TC. Reliability and validity of two Likert versions of the Schizotypal Personality Questionnaire (SPQ). *Personal Individ Differ* 2005; **38**: 1543–1548.

5 Baron-Cohen S, Wheelwright S, Skinner R, Martin J, Clubley E. The Autism-Spectrum Quotient (AQ): Evidence from Asperger Syndrome/High-Functioning Autism, Malesand Females, Scientists and Mathematicians. *J Autism Dev Disord* 2001; **31**: 5–17.

6 Wilson M. spant: An R package for magnetic resonance spectroscopy analysis. *J Open Source Softw* 2021; **6**: 3646.

7 Wilson M. Adaptive baseline fitting for MR spectroscopy analysis. *Magn Reson Med* 2021; **85**: 13–29.

8 Barkhuijsen H, de Beer R, van Ormondt D. Improved algorithm for noniterative time-domain model fitting to exponentially damped magnetic resonance signals. *J Magn Reson 1969* 1987; **73**: 553–557.

9 Ashburner J, Barnes G, Chen C-C, Daunizeau J, Flandin G, Friston K *et al.* SPM12 Manual. 2021.

10 Gasparovic C, Song T, Devier D, Bockholt HJ, Caprihan A, Mullins PG *et al.* Use of tissue water as a concentration reference for proton spectroscopic imaging. *Magn Reson Med* 2006; **55**: 1219–1226.

11 Wilson M, Andronesi O, Barker PB, Bartha R, Bizzi A, Bolan PJ *et al.* Methodological consensus on clinical proton MRS of the brain: Review and recommendations. *Magn Reson Med* 2019; **82**: 527–550.

12 Akoglu H. User’s guide to correlation coefficients. *Turk J Emerg Med* 2018; **18**: 91–93.

13 Gu Z, Gu L, Eils R, Schlesner M, Brors B. circlize implements and enhances circular visualization in R. *Bioinformatics* 2014; **30**: 2811–2812.

14 Mullins PG, Chen H, Xu J, Caprihan A, Gasparovic C. Comparative reliability of proton spectroscopy techniques designed to improve detection of J-coupled metabolites. *Magn Reson Med* 2008; **60**: 964–969.

15 Tingley D, Yamamoto T, Hirose K, Keele L, Imai K. mediation: R Package for Causal Mediation Analysis. *J Stat Softw* 2014; **59**: 1–38.

Tables

Table S1: ¹H-MRS quality parameters and Glx levels by region for Ospey+LCM

| **Region** | **ACC** | **PUT R** | **PUT L** | **DLPFC R** | **DLPFC L** |
| --- | --- | --- | --- | --- | --- |
| n | 53 | 33 | 38 | 53 | 52 |
| Glx (SD), mol/kg | 33.37 (2.07) | 33.45 (2.02) | 32.47 (2.07) | 24.42 (2.00) | 25.26 (2.44) |
| CRLB (SD), % | 5.21 (0.49) | 5.42 (1.06) | 5.68 (0.93) | 5.55 (0.57) | 5.40 (0.53) |
| tNAA (SD), mol/kg | 20.54 (0.90) | 17.78 (1.21) | 18.03 (0.83) | 20.62 (0.94) | 20.91 (1.03) |
| CRLB (SD), % | 2.60 (0.99) | 6.06 (1.87) | 5.79 (2.04) | 2.15 (0.36) | 2.15 (0.36) |
| tCr (SD), mol/kg | 17.36 (0.94) | 17.36 (0.73) | 16.49 (0.92) | 14.23 (0.86) | 14.27 (0.89) |
| CRLB (SD), % | 2.00 (0.00) | 2.33 (0.48) | 2.58 (0.50) | 1.98 (0.14) | 2.00 (0.28) |
| *Note: Values are mean (SD)*  *Glx, glutamate+glutamine;* *ACC, anterior cingulate cortex; PUT R, right putamen; PUT L, left putamen; DLPFC R, right dorsolateral prefrontal cortex; DLPFC L, left dorsolateral prefrontal cortex; Glu, Glutamate; CRLB, Cramer-Rao Lower Bound; SD, standard deviation* | | | | | |

Table S2: ¹H-MRS quality parameters and metabolite levels by region for Spant+ABfit

| **Region** | **ACC** | **PUT R** | **PUT L** | **DLPFC R** | **DLPFC L** |
| --- | --- | --- | --- | --- | --- |
| n | 53 | 50 | 52 | 53 | 51 |
| Glu (SD), mol/kg | 18.18 (2.95) | 16.49 (2.02) | 17.12 (2.00) | 13.01 (1.86) | 13.67 (2.90) |
| CRLB (SD), % | 3.70 (0.64) | 6.01 (1.11) | 5.72 (0.95) | 4.07 (0.68) | 4.01 (0.83) |
| tNAA lw (SD), ppm | 0.04 (0.00) | 0.07 (0.01) | 0.07 (0.01) | 0.05 (0.01) | 0.05 (0.01) |
| SNR | 148.02 (18.33) | 57.32 (5.91) | 59.78 (7.71) | 207.65 (30.95) | 202.89 (29.51) |
| *Note: Values are mean (SD)*  *Glu, glutamate; tNAA, total-N-acetylaspartate (NAA, N-acetylaspartate + NAAG, N-acetyl-aspartylglutamate); ACC, anterior cingulate cortex; PUT R, right putamen; PUT L, left putamen; DLPFC R, right dorsolateral prefrontal cortex; DLPFC L, left dorsolateral prefrontal cortex;* *CRLB, Cramer-Rao Lower Bound; tNAA lw, tNAA linewidth; SNR, Signal-to-noise Ratio; SD, standard deviation* | | | | | |

Table S3: Group differences SPQ and AQ

|  | **Low SPQ** | **High SPQ** | **P-value**^1^ | **W** |
| --- | --- | --- | --- | --- |
| Age | 23.59 (3.91) | 23.65 (3.90) | 0.9857 | 349.5 |
| SPQ: total score (/296) | 49.59 (15.75) | 133.00 (32.67) | <0.0001 | 0 |
| AQ: total score (/50) | 18.66 (6.92) | 24.04 (6.70) | 0.0084 | 202.5 |
| *Note: Median split for psychotic-like experiences, Median SPQ = 75; Low SPQ, SPQ<=75; High SPQ, SPQ>75; SPQ, Schizotypal Personality Questionnaire; Values are mean (SD)* | | | | |

|  | **Low AQ** | **High AQ** | **P-value**^1^ | **W** |
| --- | --- | --- | --- | --- |
| Age | 22.67 (2.80) | 24.62 (4.58) | 0.1464 | 269.5 |
| SPQ: total score (/296) | 70.67 (36.50) | 111.12 (52.52) | 0.0039 | 188.5 |
| AQ: total score (/50) | 14.37 (4.28) | 27.46 (3.68) | <0.0001 | 0 |
| *Note: Median split for autistic traits, Median AQ = 22; Low AQ, SPQ<=22; High AQ, AQ>22; AQ, Autism Spectrum Quotient; Values are mean (SD)* | | | | |

^1^Wilcoxon rank sum test

**Figures**

*Figure S1: Distribution of the clinical scores*


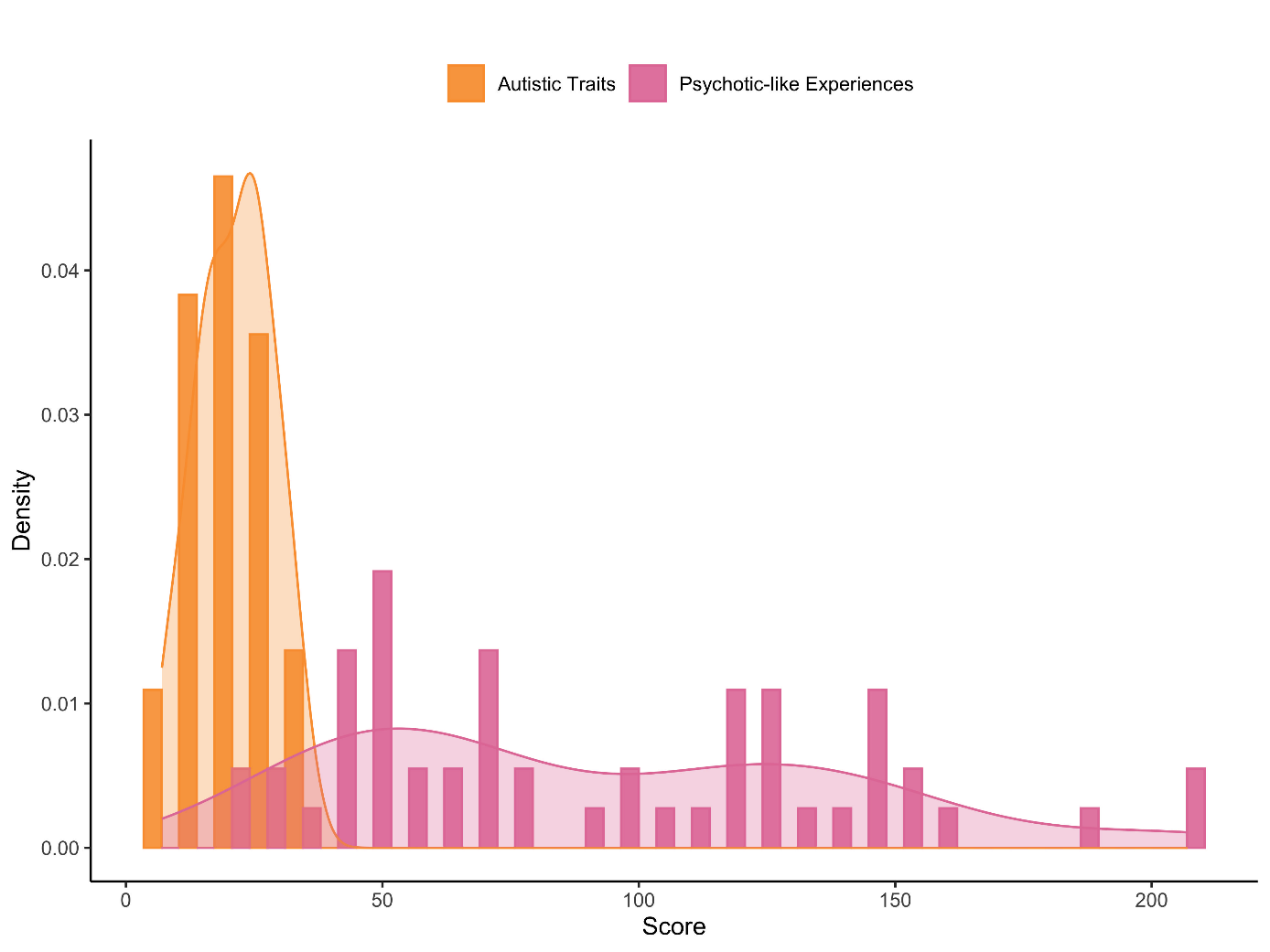


*Note:* *The histogram shows the distribution of the autistic traits (AQ) and the psychotic-like experiences (SPQ) marked by the different colours for n=53. The SPQ has a maximum score of 296 and the AQ of 50 points.*

Figure S2: MRS-data Processing and Analysis workflow

*
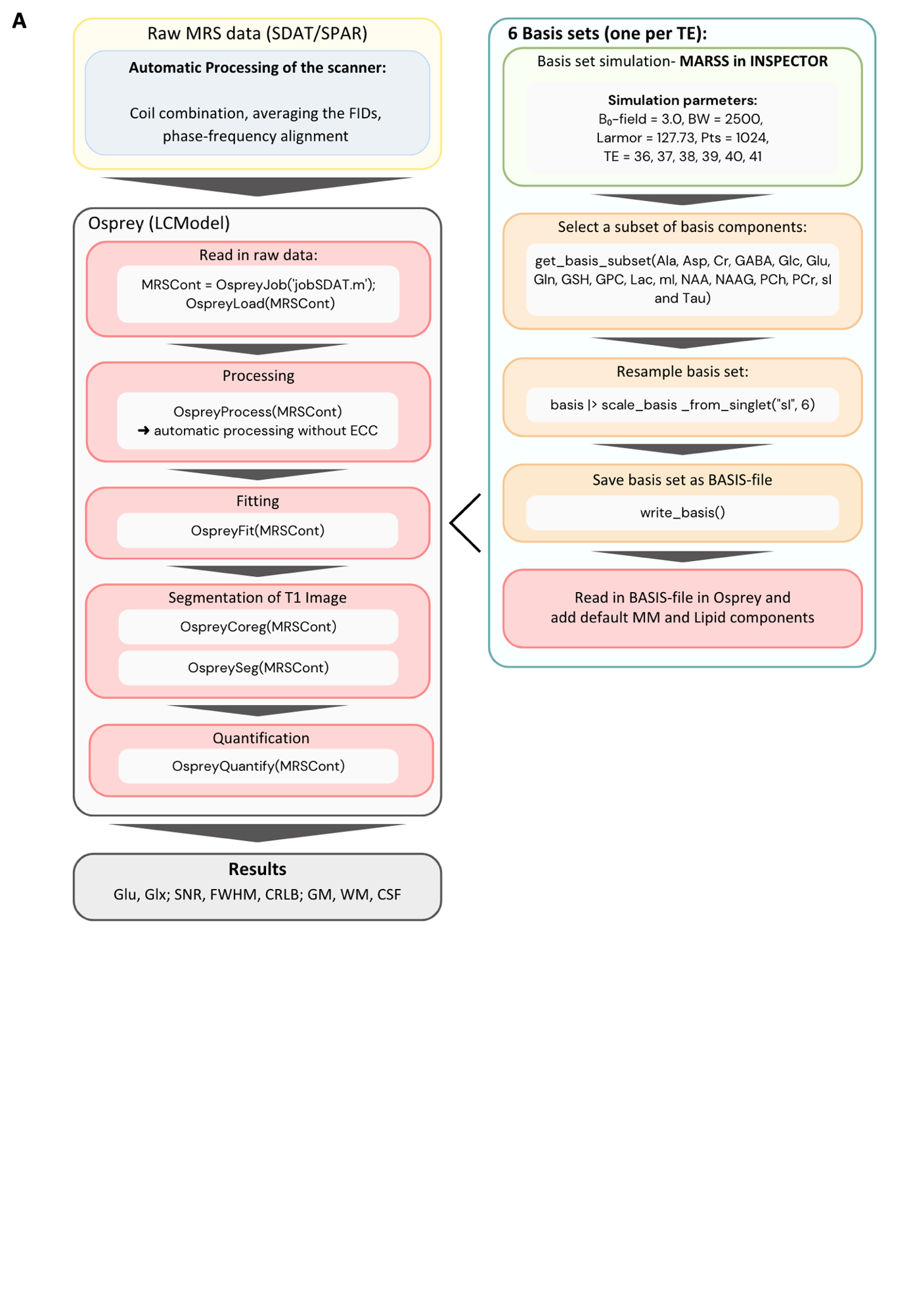
*

*
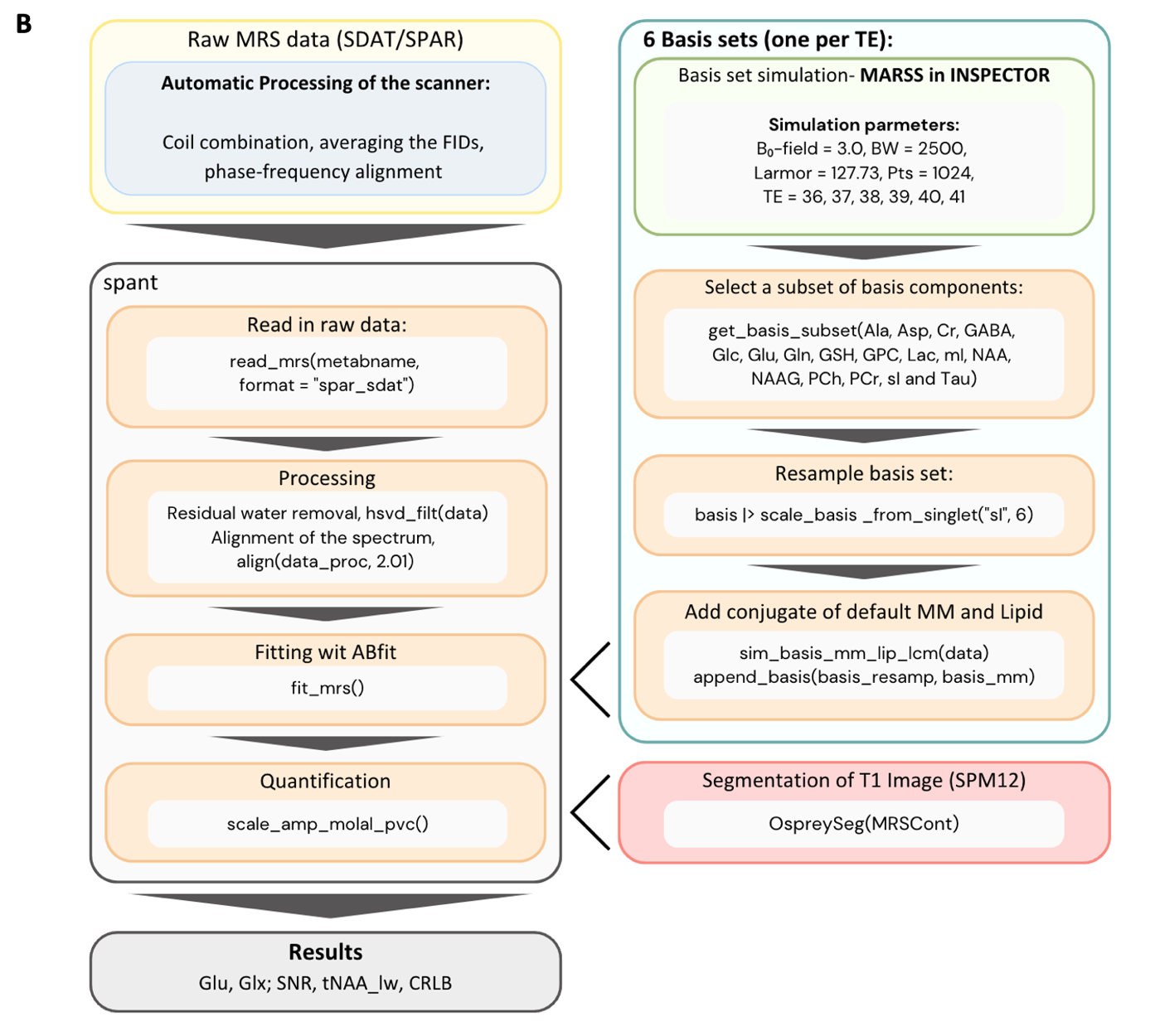
Note: Workflow of our MRS-data processing for A) Osprey + LCM and B) spant+ ABfit, Colors indicate the different used tools; green for MARSS in INSPECTOR, red for Osprey and orange for spant;* *BW, bandwidth; TE, time of echo; MM, makromolecules; Ala, alanine; Asp, aspartate; Cr, creatine; GABA; Glc, glucose; Glu, glutamate; Gln, glutamine; Glx, Glu + Gln; GSH, glutathione; GPC, glycerophosphocholine; Lac, lactate; mI, myoinosito; NAA, N-acetylaspartate; NAAG, N-acetyl-aspartylglutamate; PCh, phosphocholine; PCr, phosphocreatine; sI, scyllo-inositol; Tau, taurine; SNR, Signal-to-noise Ratio; tNAA, NAA + NAAG; lw, linewidth; CRLB, Cramer-Rao Lower Bound; FWHM, full width at half maximum, measurement for the water linewidth; GM, grey matter; WM, white matter; CSF, Cerebrospinal fluid*

*Figure S3:* Representative fitted MRS spectra

*
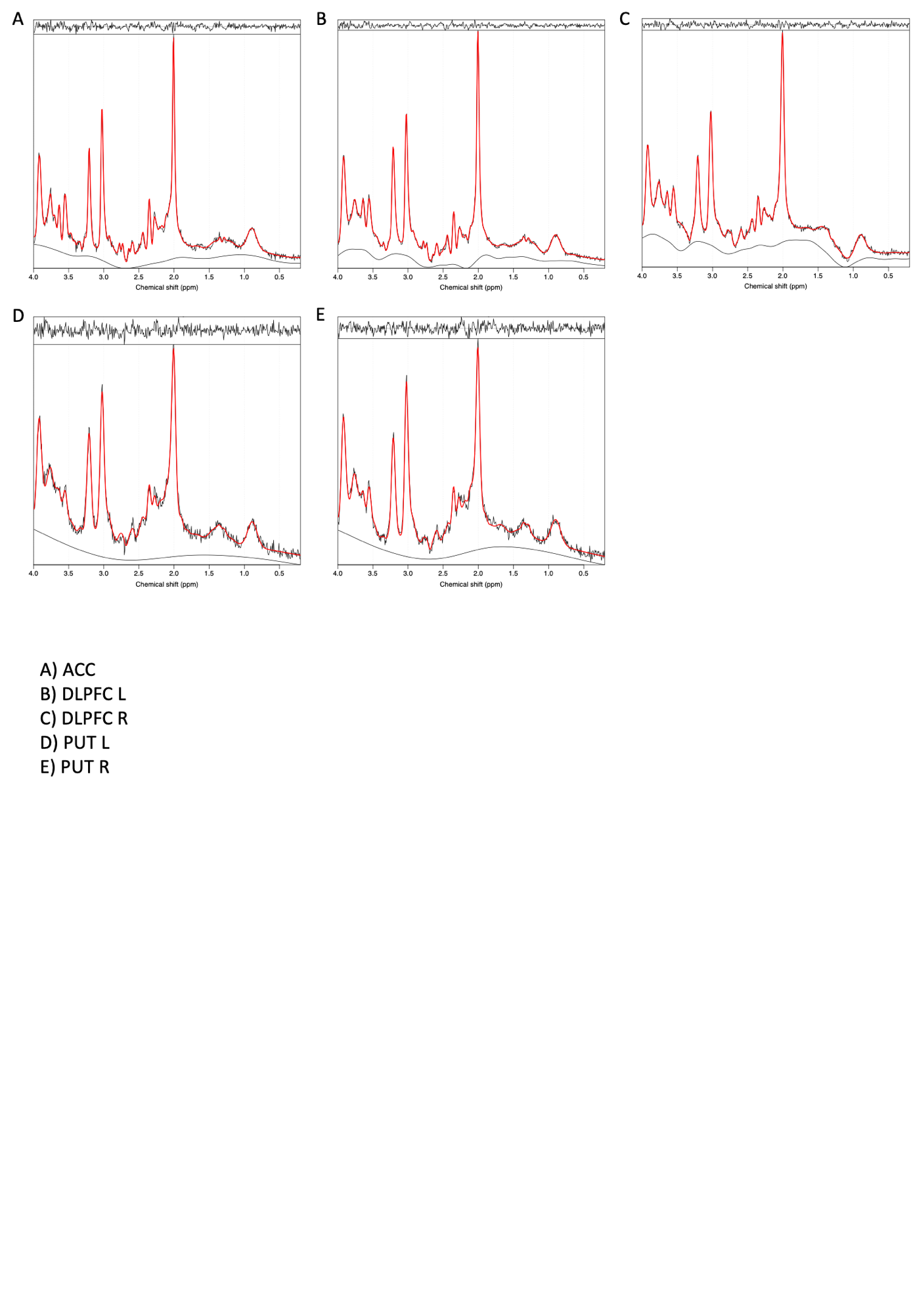
*

*Note: ^1^H-MRS spectrum fitted by spant+ABfit of the (A) ACC, (B) DLPFC left, (C) DLPFC right, (D) putamen left, and (E) putamen right. ACC, anterior cingulate cortex; PUT R, right putamen; PUT L, left putamen; DLPFC R, right dorsolateral prefrontal cortex; DLPFC L, left dorsolateral prefrontal cortex.*

*Figure S4: Visualization of the correlations between the subscores*

*
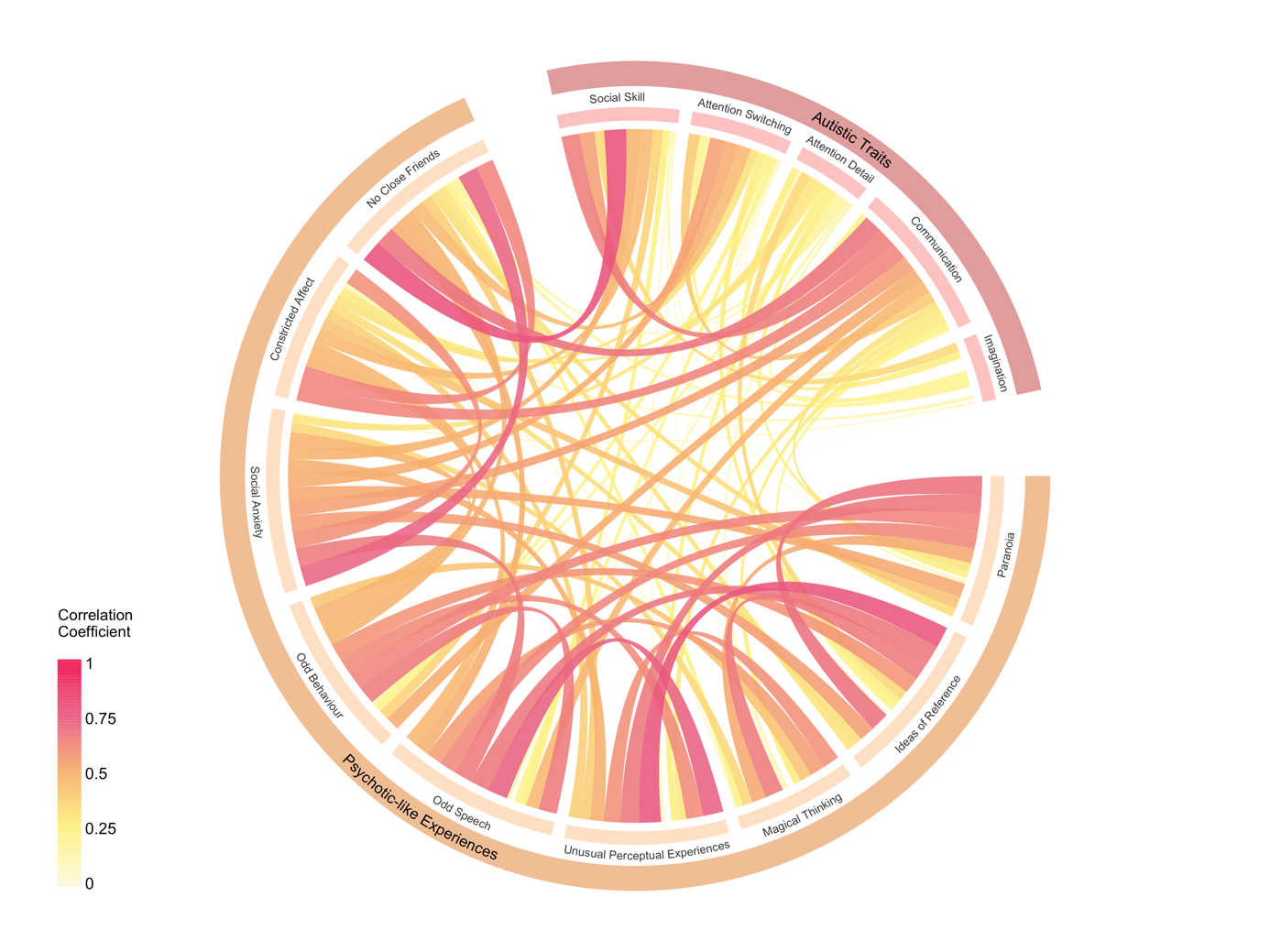
*

*Note: The Chord Diagram describes the relationship between the individual items of the AQ and SPQ using Spearman Correlation Coefficients. The subscores are structured according to their total scores (i.e., Autistic Traits for AQ; Psychotic-like Experiences for SPQ). The color and width of the bidirectional links indicate the strength of the connection between each of the subscores – the wider and darker the stronger.*
